# Dosimetric Comparison of Noncoplanar VMAT Without Rotating the Patient Couch Versus Conventional Coplanar/Noncoplanar VMAT for Head and Neck Cancer: First Report of Dynamic Swing Arc

**DOI:** 10.1016/j.adro.2024.101706

**Published:** 2024-12-30

**Authors:** Kouta Hirotaki, Kento Tomizawa, Satoe Kitou, Shunta Jinno, Shunsuke Moriya, Takeshi Fujisawa, Sadamoto Zenda, Takeji Sakae, Masashi Ito

**Affiliations:** a Doctoral Program in Medical Sciences, Graduate School of Comprehensive Human Sciences, University of Tsukuba, Ibaraki, Japan; b Department of Radiological Technology, National Cancer Center Hospital East, Chiba, Japan; c Department of Radiation Oncology, National Cancer Center Hospital East, Kashiwa, Japan; d Healthcare Business Group, Hitachi High-Tech Corporation, Tokyo, Japan; e Faculty of Medicine, University of Tsukuba, Ibaraki, Japan; f Division of Radiation Oncology and Particle Therapy, Exploratory Oncology Research and Clinical Trial Center, National Cancer Center, Kashiwa, Japan

## Abstract

**Purpose:**

This retrospective planning study aimed to verify the usefulness of a clinically available method, dynamic swing arc (DSA), a noncoplanar volumetric modulated arc therapy (VMAT) technique, of the new accelerator OXRAY for head and neck squamous cell carcinoma (HNSCC). We performed dosimetric comparisons between DSA and conventional coplanar/noncoplanar VMAT (C-VMAT/NC-VMAT) plans for HNSCC.

**Methods and Materials:**

We selected 32 patients with oropharyngeal and hypopharyngeal cancer treated with C-VMAT at National Cancer Center Hospital East between September 2018 and July 2023. DSA and C/NC-VMAT plans were generated using OXRAY and TrueBeam, respectively. DSA employed noncoplanar 2-arc beams with an O-ring gantry swing, whereas C-VMAT and NC-VMAT used coplanar and noncoplanar 2-arc beams, respectively. Dosimetric parameters, normal tissue complication probability, and delivery times were compared pairwise using the Wilcoxon signed-rank test with Bonferroni correction.

**Results:**

For high-risk planning target volume (PTV), D98 values in NC-VMAT plans were closest to the prescribed dose, significantly differing from C-VMAT and DSA plans. DSA plans showed significantly better median conformity and homogeneity indices (0.97 and 7.33, respectively) compared to C-VMAT (0.95 and 8.36) and NC-VMAT (0.96 and 7.96) plans. DSA plans significantly reduced the mean ipsilateral/contralateral parotid gland dose by 5.78/6.93 and 2.88/1.56 Gy (median) compared to C-VMAT and NC-VMAT. NC-VMAT and DSA plans significantly decreased the mean oral cavity dose by 2.16 and 3.22 Gy (median) compared to C-VMAT. DSA plans had the lowest median normal tissue complication for xerostomia with significant differences, followed by NC-VMAT and C-VMAT. The delivery time for DSA plans was longer than VMAT (151 seconds vs 124 seconds), but shorter than NC-VMAT.

**Conclusions:**

DSA plans using OXRAY for HNSCC maintained PTV coverage while reducing parotid gland and oral cavity mean doses compared to coplanar VMAT plans, although delivery times increased. DSA plans reduced parotid gland doses and delivery times compared to noncoplanar VMAT plans.

## Introduction

Head and neck squamous cell carcinoma (HNSCC) is the seventh most common cancer worldwide.^1^ Radiation therapy (RT) is widely used in the management of HNSCC, since it provides both effective tumor control and functional preservation.^2^ In RT for HNSCC, the complex anatomy of the head and neck exposes patients to various types of radiation-induced toxicity, including xerostomia, oral mucositis, and myelitis.^3^

RT techniques such as intensity-modulated RT (IMRT) have been widely used to reduce radiation-induced toxicities in patients with HNSCC.^4^^,^^5^ However, even when IMRT is used for HNSCC, the target is often close to the surrounding organs at risk (OARs), which does not always provide an ideal dose distribution. A phase 3, multicenter, randomized controlled trial reported that 71% of patients with HNSCC treated using IMRT experienced grade ≥ 2 acute xerostomia.^6^ Another randomized controlled trial using IMRT for patients with HNSCC reported that the grade ≥ 2 late xerostomia rate was 23%.^7^ Therefore, there is a need for new irradiation techniques to reduce OAR doses while maintaining target doses compared to conventional IMRT. To address this issue, noncoplanar irradiation using multiple arcs at different static couch angles,^8^^,^^9^ and dynamic trajectory RT using simultaneous gantry and couch rotation during beam-on have been developed.^10^^,^^11^ However, these irradiation methods require couch rotation, which may increase the treatment time, patient burden, and risk of patient position error compared with conventional coplanar irradiation.

Recently, the use of noncoplanar irradiation with an O-ring gantry swing without moving the couch has been explored. This method is expected to provide better dose distribution than conventional coplanar IMRT. Vero4DRT (Mitsubishi Heavy Industries Ltd) is a pioneering linear accelerator that combines noncoplanar irradiation using a swingable O-ring gantry with volumetric modulated arc therapy (VMAT).^12^^,^^13^ However, owing to its relatively small field size, Vero4DRT is unsuitable for cancers requiring large-field irradiation, including the head and neck region.

OXRAY (Hitachi, Ltd) is a novel linear accelerator with a swingable O-ring gantry and a larger field size than Vero4DRT (https://www.nature.com/articles/d42473-023-00445-6). Dynamic swing arc (DSA) is a noncoplanar VMAT technique specific to OXRAY, based on the O-ring gantry swing synchronized with the gantry rotation, leading to a flexible trajectory. OXRAY is also equipped with a short-width, high-speed drive multileaf collimator (MLC, 2.5-mm width at the center), which has the potential to address the challenges of anatomic complexity in the head and neck region. Therefore, the usefulness of OXRAY planning in HNSCC must be verified. However, to the best of our knowledge, there have been no planning studies using DSA for HNSCC treatment.

We performed a retrospective planning study to compare dosimetric profiles between plans using the DSA technique with OXRAY and conventional coplanar/noncoplanar VMAT for HNSCC.

## Methods and Materials

### Patients and imaging data sets

Thirty-two patients with oropharyngeal and hypopharyngeal cancers treated using conventional coplanar VMAT (C-VMAT) in Natinal Cancer Center Hospital East between September 2018 and July 2023 were randomly selected from medical records (Table 1).^14^ All patients were immobilized using a 4 or 5-point thermoplastic mask covering the shoulder and a patient-specific pillow. Simulation computed tomography (2-mm slices) data sets were acquired using an Aquilion One scanner (Canon Medical Systems). Although the requirement for informed consent was waived owing to the retrospective nature of the study, the details of this study were published on the center's homepage and patients were allowed to refuse to participate in the study. The contents of this study, including the retrospective investigation procedure and handling of patient information, were approved by the Institutional Review Board of National Cancer Center Hospital East (approval number: 2018-076).

**Table 1:** Patient characteristics

Patient	Age, y	Sex	TNM*	High-risk CTV volume (cm^3^)	Site
1	78	Male	T4N3M0	157.83	Oropharynx
2	76	Male	T2N1M0	57.68	Oropharynx
3	80	Male	T2N3M0	190.31	Oropharynx
4	74	Male	T4N1M0	154.55	Oropharynx
5	69	Male	T2N2M0	99.75	Oropharynx
6	73	Male	T4N0M0	84.08	Hypopharynx
7	71	Male	T1N3M0	211.23	Hypopharynx
8	52	Male	T2N1M0	27.76	Oropharynx
9	51	Male	T2N3M0	404.98	Oropharynx
10	81	Male	T2N2M0	116.08	Oropharynx
11	63	Male	T2N1M0	63.73	Hypopharynx
12	60	Female	T4N2M0	98.11	Oropharynx
13	80	Male	T4N1M0	156.22	Oropharynx
14	42	Male	T1N3M0	897.52	Oropharynx
15	41	Male	T4N2M0	182.87	Hypopharynx
16	66	Male	T2N2M0	53.63	Hypopharynx
17	74	Male	T2N2M0	122.12	Hypopharynx
18	69	Male	T2N3M0	277.02	Hypopharynx
19	71	Male	T2N3M0	158.57	Hypopharynx
20	61	Male	T4N3M0	94.23	Hypopharynx
21	71	Male	T1N2M0	76.07	Hypopharynx
22	54	Male	T4N2M0	341.03	Oropharynx
23	74	Male	T1N2M0	165.03	Oropharynx
24	63	Male	T2N0M0	72.69	Oropharynx
25	68	Male	T4N1M0	174.76	Oropharynx
26	65	Female	T4N2M1	150.11	Oropharynx
27	55	Male	T4N2M1	44.37	Oropharynx
28	80	Male	T4N2M0	91.2	Oropharynx
29	42	Male	TXN3M0	164.3	Oropharynx
30	69	Male	T2N2M0	135	Oropharynx
31	57	Male	T4N2M0	162.22	Oropharynx
32	72	Female	T4N1M0	134.34	Oropharynx

*Abbreviation:* CTV = clinical target volume.

⁎ UICC TNM Classification of Malignant Tumours, 8th edition.^14^

### Mechanical configuration of TrueBeam and OXRAY

We adopted TrueBeam (Varian Medical Systems) to compare DSA plans using OXRAY with conventional C-VMAT/noncoplanar VMAT (NC-VMAT) plans. Table 2 shows a comparison of the mechanical configurations of TrueBeam and OXRAY. The MLC thickness of OXRAY is 110 mm, and it has superior shielding capabilities compared to TrueBeam (67 mm). The TrueBeam and OXRAY MLC transmissions measured at our institute were 0.01520 and 0.00237, respectively. The MLC drive speed is 6.50 cm/second, which is about 3 times faster than that of TrueBeam (2.50 cm/second). OXRAY features a clinically available DSA technique that swings the O-ring frame during irradiation, enabling noncoplanar irradiation without moving the patient's couch. The O-ring could rotate up to 60°, and its swing angle, direction of ring rotation, and trajectory modulation points can be specified by the user. OXRAY has a dual-kV cone beam computed tomography system, consisting of dual radiograph tubes that are equipped at orthogonal angles to each other, allowing kV images to be acquired from 2 directions simultaneously. Also, OXRAY detects collisions and the gantry movement stops when it comes into contact with the patient or couch. Additionally, the system has a noncontact collision prevention system that warns against movement within the collision area.

**Table 2:** Mechanical configurations of TrueBeam and OXRAY

	TrueBeam	OXRAY
MLC type	Millennium 120	-
MLC width (central) [mm]	5.00	2.50
MLC width (peripheral) [mm]	10.00	5.00
MLC thickness [mm]	67.00	110.00
Maximum leaf speed [cm/s]	2.50	6.50
Maximum leaf difference [cm]	15.00	22.05
Minimum dynamic leaf gap [cm]	0.05	0.20
Maximum gantry speed variation [°/s]	0.75	0.75
Minimum gantry speed [°/sec]	0.50	0.10
Maximum gantry speed [°/sec]	6.00	7.00
Supported trajectory ring angle [°]	-	300/60
Maximum ring angle speed [°/s]	-	5.50
Minimum ring angle speed [°/s]	-	0.10
Dose rate	Variable	Variable
Minimum dose rate [MU/min]	10.00	150.00
Maximum dose rate [MU/min]	600.00	550.00

*Abbreviations:* MLC = multileaf collimator; MU = monitor unit.

### Contouring

Radiation oncologists delineated the planning targets and OARs. Gross tumor volumes of the primary tumor and any lymph node metastases were localized, and 10-mm margins were added to construct the high-risk clinical target volume (CTV). The low-risk CTV included the relevant regional lymph nodes. An expansion with a margin of 0.5 cm was given in all directions for high- and low-risk CTV to create the planning target volume (PTV). OARs include the spinal cord, brain, brainstem, eyes, lenses, cochlea, inner ears, oral cavity, parotid glands, larynx, mandible, pharyngeal constrictor muscle (PCM), brachial plexus, and lung apexes.

### Treatment planning and clinical goal

The clinical goals of the treatment planning are listed in Table 3. The target regions received 2 dose levels in 35 fractions, with a simultaneous integrated boost. The prescribed doses were 70 Gy and 56 Gy for the high- and low-risk CTV, respectively. When the clinical goals were achieved, the radiation dose was reduced to the parotid glands and oral cavity as much as possible. If all clinical goals were difficult to achieve, plans were created with the highest priority given to achieving the dose constraints for the spinal cord and high-risk PTV. To improve the robustness of dose distribution against shoulder setup errors during treatment, the “protect function” in Raystation was used. This function shields the beam passing through the shoulder using the MLC. The region of interest for the shoulder was automatically generated using Raystation's scripting function.

**Table 3:** Clinical goals for treatment planning

Structure	Constraint
High-risk PTV	D95% > 98% prescribed dose
Low-risk PTV	D95% > 97% prescribed dose
Spinal cord	Maximum dose < 45 Gy
Brainstem	Maximum dose < 54 Gy
Oral cavity	Mean dose < 30 Gy
Parotid gland	Mean dose < 26 Gy (at least 1 side)
Cochlea	Mean dose < 40 Gy
Larynx	Mean dose < 45 Gy
Body	Maximum dose < 107% prescribed dose

*Abbreviations:* DX% = dose received by X% of the structure's volume; PTV = planning target volume.

### VMAT plan

All conventional C-VMAT plans have been delivered in clinical practice. Dosimetrists generated these plans using the RayStation treatment planning system (RaySearch Laboratories AB) with a collapsed cone-convolution algorithm. The C-VMAT plans were created using 6-mV photon beams with 2 full arcs (counterclockwise rotation from 179° to 181° and clockwise rotation backward) and collimator angles of 350° and 10°, respectively. Three arcs and arbitrary collimator angles were used for large targets, where it was difficult to achieve the clinical goal using 2 arcs. All simulated NC-VMAT plans were created by a dosimetrist using RayStation. The NC-VMAT plans were created using 6-mV photon beams with 2 full arcs (counterclockwise rotation from 179° to 181° and clockwise rotation backward), couch angles of 20° and 340°, and collimator angles of 355° and 5°, respectively.

### DSA plan

**Figure 1:**
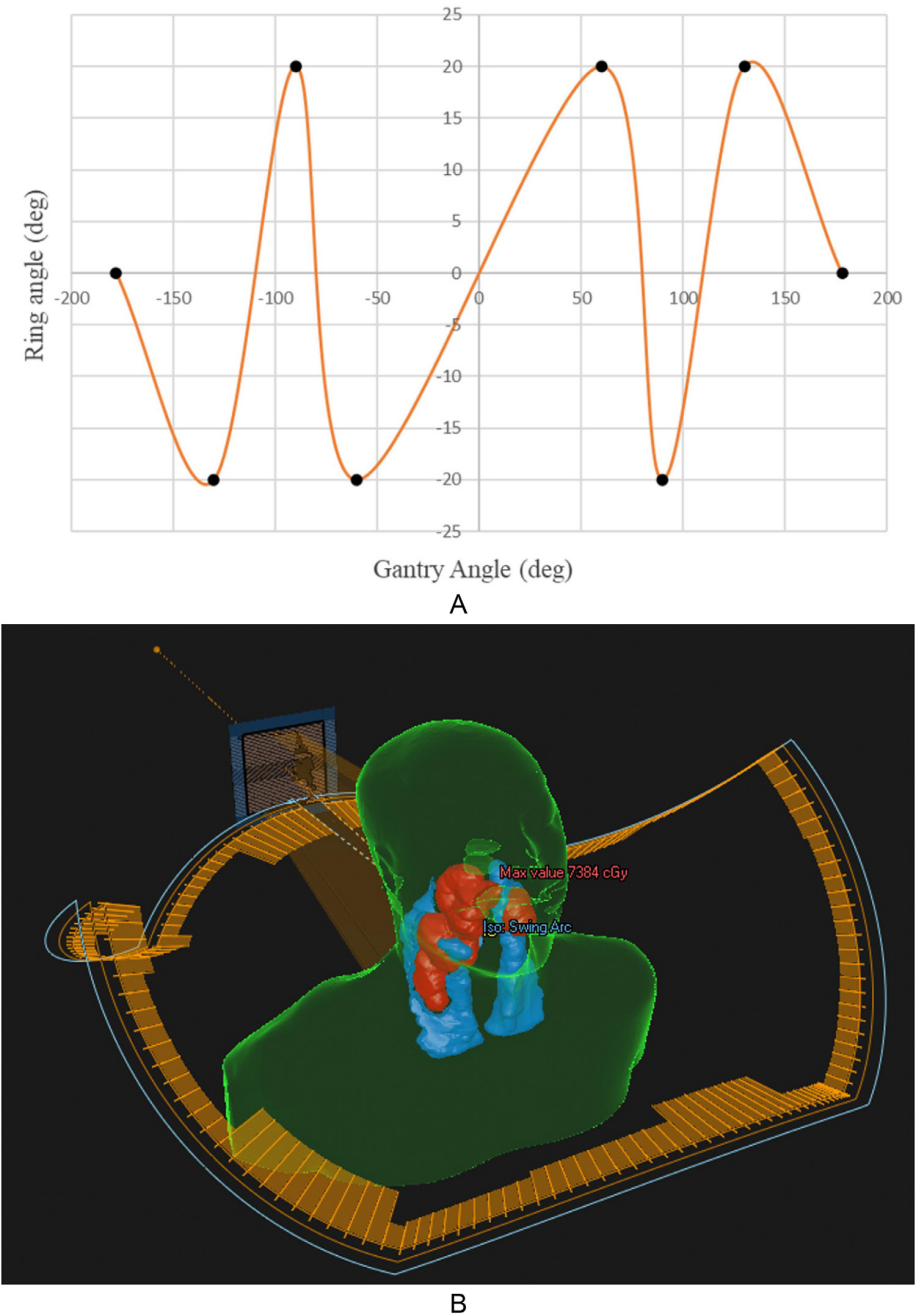
The change in O-ring angle synchronized with the gantry angle using a dynamic swing arc (A) and its arc trajectory (B). The black dots are the modulation points of the gantry trajectory and specify the gantry angle and ring angle to be reached at the next modulation point.

All DSA simulation plans were generated by a dosimetrist using a RayStation. We adopted 6-mV photon beams in the DSA plans. The ring angles were set between 340° and 20°, and the O-ring continuously swung in synchrony with the gantry rotation. Modulation was performed in 6 sections per arc to avoid the shoulders and OARs. The O-ring angle and modulation points were manually selected by a dosimetrist to minimize the overlap between the PTVs and OARs while avoiding O-ring and couch collisions. During a single-arc gantry orbit from 178° to 182°, ring swing was modulated (Fig. 1A, B).

### Dosimetric evaluation

For the planning targets, high-risk PTV D98% (DX, dose received by X% of the structural volume), D50%, D2%, conformity index (CI), homogeneity index (HI), and low-risk PTV D98% were evaluated. For the overdose region, the body maximum point dose (Dmax), and V105% (VX, percentage of the volume that received X% or more of the prescribed dose) were evaluated. For the OARs, the mean dose to the parotid glands, spinal cord Dmax, oral cavity mean dose, and mandible D1cc (dose received by a volume of 1 cc) were evaluated. CI and HI are defined in (1), (2), respectively, as follows:

(1) CI=(TV_PIV)²/(TV×PIV)

TV_PIV represents the target volume encompassed by the prescribed isodose volume (PIV), where TV denotes the target volume, and PIV refers to the volume enveloped by the prescribed isodose.

(2) HI=(D2%−D98%)/prescribedose×100

Delivery times and total monitor unit (MU) values were compared. The delivery time was calculated using RayStation based on elements such as gantry rotation, DSA modulation angle, and dose rate, and did not include the time of patient setup, imaging, and registration in image-guided RT. In this study, the calculated delivery time included the maximum speed of the components and also considered acceleration and deceleration. The treatment time was calculated from the gantry speed and the dose rate at each control point. In the NC-VMAT plans, couch movement time was measured and added to the treatment time.

To estimate the impact of dose differences on clinical complications, normal tissue complication (NTCP), including xerostomia (parotid) and dysphagia (PCM) were calculated using the Lyman–Kutcher–Burman model for all plans.^15^^,^^16^ The parameters used for NTCP calculation were taken from a previous study (Table E1).

### Statistics

For dosimetric, delivery time, and NTCP comparisons between the 3 plans, pairwise comparisons were performed using the Wilcoxon signed-rank test with Bonferroni correction. Statistical significance was defined as a 2-tailed *P* value < .05. All analyses were performed using R version 4.2.2 (R Foundation for Statistical Computing).

## Results

### Target volume coverage

**Figure 2:**
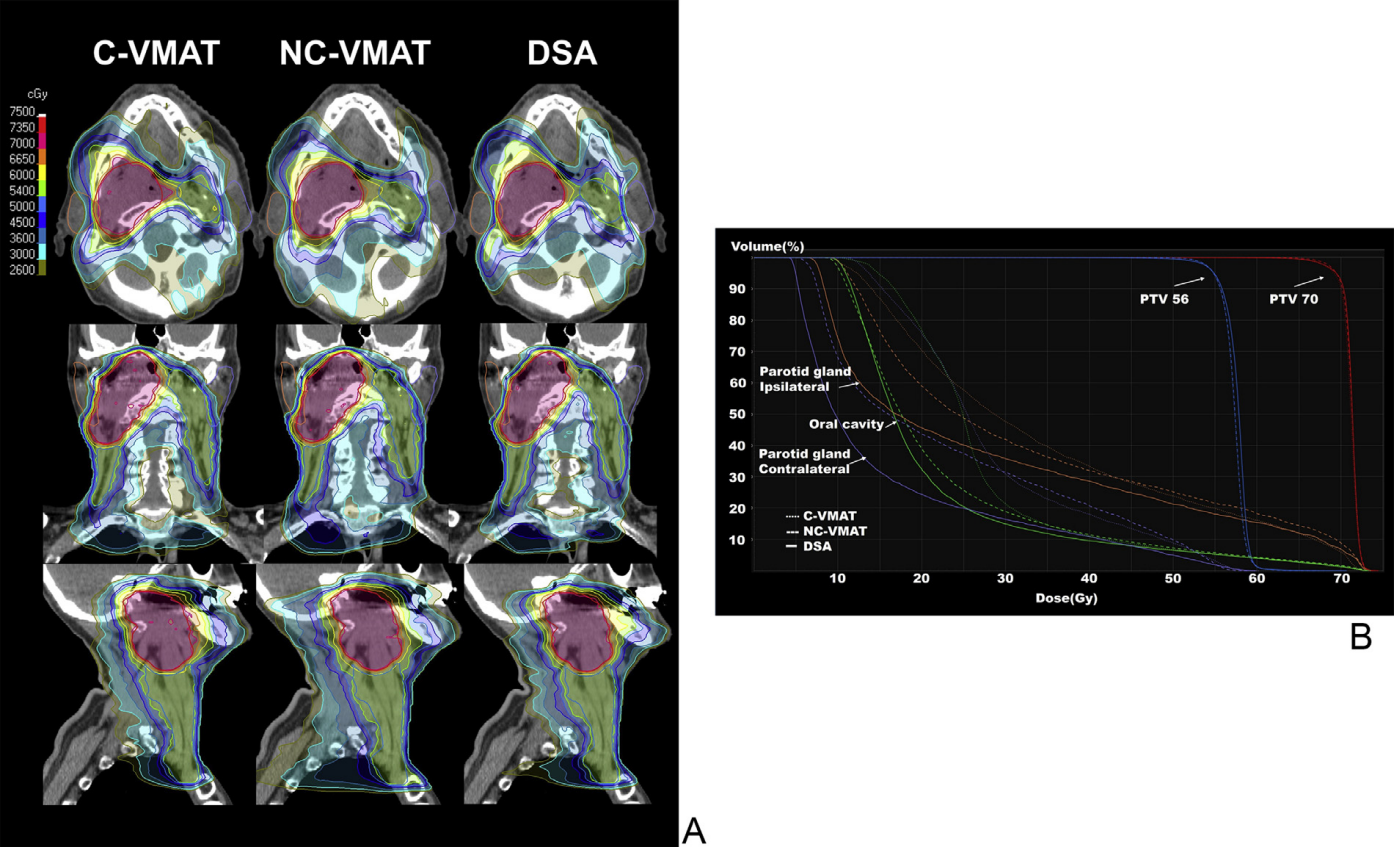
Representative case comparison of dose distribution (A) and dose-volume histogram (B) between conventional coplanar volumetric modulated arc therapy (C-VMAT), noncoplanar VMAT (NC-VMAT), and dynamic swing arc (DSA) plans. High- and low-risk PTVs are represented in pink and blue, respectively. *Abbreviation:* PTV = planning target volume.

Figure 2 shows representative cases comparing the 3 plans. A comparison of the dose indices for PTV is presented in Table 4. In the dose coverage indices of high-risk PTV, D98 values in the NC-VMAT plans were the closest to the prescribed dose, with significant differences compared with the C-VMAT and DSA plans (*P* < .001 and *P* < .001, respectively). On the other hand, D50 values in the DSA plans were the highest, with significant differences compared with the NC-VMAT plans (*P* < .001). For D98 and D50, there were no significant differences between the DSA and C-VMAT plans (*P* = .342 and *P* = 1.000, respectively). D2 values in the DSA plans were closer to the prescribed dose, with a significant difference from those of the C- and NC-VMAT plans (*P* < .001 and *P* < .001, respectively). DSA plans had the best CI, with significant differences from the C-VMAT and NC-VMAT plans (*P* < .001 and *P* < .001, respectively). There was no significant difference in CI values between the C-VMAT and NC-VMAT plans (*P* = .316). For HI, DSA plans were the best, followed by NC-VMAT and C-VMAT plans, with significant differences (*P* < .001 and *P* < .001, respectively). Additionally, the HI values in the NC-VMAT plans were better than those in the C-VMAT plans (*P* = .009). Regarding the low-risk PTV coverage index, the D98 of the DSA plans was the best, with significant differences from those of the C-VMAT and NC-VMAT plans (*P* = .015 and *P* < 0.001, respectively). D98 values in the NC-VMAT plans were closer to the prescribed dose than those in the C-VMAT plans (*P* < .001).

**Table 4:** Comparison dose indices, delivery time, and total MU between coplanar/noncoplanar VMAT and DSA plans

	C-VMAT	NC-VMAT	DSA	*P* values*		
	Median (IQR)	Median (IQR)	Median (IQR)	C-VMAT vs NC-VMAT	C-VMAT vs DSA	NC-VMAT vs DSA
High-risk PTV						
D98 (%)	96.26 (94.93-97.33)	96.85 (95.80-97.67)	96.71 (95.76-97.15)	<.001	.342	<.001
D50 (%)	102.00 (101.83-102.15)	101.95 (101.89-102.10)	102.01 (101.89-102.13)	<.001	1.000	<.001
D2 (%)	104.53 (104.14-104.66)	104.38 (104.22-104.75)	104.04 (103.84-104.16)	<.001	<.001	<.001
CI	0.95 (0.93-0.96)	0.96 (0.94-0.97)	0.97 (0.96-0.98)	.316	<.001	<.001
HI	8.36 (7.16-9.33)	7.96 (6.83-8.64)	7.33 (6.74-8.29)	<.001	.009	<.001
Low-risk PTV						
D98 (%)	95.68 (94.33-96.86)	95.69 (94.37-96.39)	96.54 (95.30-97.49)	<.001	.015	<.001
Brain						
Dmax (Gy)	51.45 (44.27-56.07)	52.00 (47.52-54.85)	50.03 (45.76-52.92)	.535	1.00	.088
Brain stem						
Dmax (Gy)	36.24 (29.58-43.07)	41.37 (35.84-47.18)	36.38 (33.89-42.79)	.001	.054	.005
Eye right						
Dmax (Gy)	1.26 (0.96-1.60)	1.99 (1.36-3.92)	1.67 (1.36-2.62)	<.001	<.001	.800
Eye left						
Dmax (Gy)	1.23(0.88-1.62)	2.29 (1.57-4.93)	1.68 (1.28-2.24)	<.001	<.001	.001
Lens right						
Dmax (Gy)	0.88 (0.70-1.04)	1.14 (0.81-1.50)	1.05 (0.88-1.34)	<.001	<.001	1.000
Lens left						
Dmax (Gy)	0.85 (0.67-0.96)	1.08 (0.81-1.47)	1.03 (0.83-1.35)	<.001	<.001	.113
Inner ear right						
Dmax (Gy)	41.71 (16.93-57.97)	46.20 (17.27-57.77)	38.34 (9.99-57.22)	1.000	.063	.034
Inner ear left						
Dmax (Gy)	39.36 (18.89-52.69)	41.63 (20.58-53.77)	31.99 (12.75-50.66)	.209	.311	<.001
Cochlea right						
Dmean (Gy)	11.32 (5.69-20.40)	9.62 (7.25-19.23)	7.41 (4.17-14.99)	1.000	<.001	<.001
Cochlea left						
Dmean (Gy)	11.72 (5.59-18.37)	15.40 (9.56-21.05)	7.62 (5.37-15.24)	.021	.005	<.001
Parotid ipsilateral						
Dmean (Gy)	29.57 (26.49-35.06)	26.67 (21.93-29.70)	23.79 (17.71-27.59)	<.001	<.001	<.001
Parotid contralateral						
Dmean (Gy)	24.42 (22.45-28.24)	19.05 (14.72-23.98)	17.49 (12.73-20.19)	<.001	<.001	<.001
Oral cavity						
Dmean (Gy)	27.53 (23.17-36.10)	25.37 (17.10-31.27)	24.31 (16.31-29.25)	<.001	<.001	.181
Mandible						
D1cc (Gy)	69.83 (61.94-71.25)	70.01 (62.91-71.44)	69.46 (65.28-71.34)	1.000	1.000	1.000
Pharyngeal constrictor muscle						
Dmean (Gy)	57.01 (55.01-59.99)	58.44 (54.98-60.78)	57.98 (54.18-59.96)	.166	1.000	<.001
Brachial plexus right						
Dmax (Gy)	60.62 (58.75-71.99)	59.59 (58.61-71.20)	59.35 (57.68-71.27)	.192	.056	1.000
Brachial plexus left						
Dmax (Gy)	59.32 (57.49-63.20)	58.81 (57.26-63.89)	58.78 (57.32-61.72)	1.000	.106	.716
Lung apex right						
Dmean (Gy)	3.50 (2.05-5.08)	4.52 (3.47-6.36)	3.68 (2.36-4.85)	<.001	1.000	<.001
Lung apex left						
Dmean (Gy)	3.59 (2.67-4.18)	4.93 (3.72-6.23)	3.89 (2.89-4.54)	<.001	.002	<.001
Spinal cord						
D1cc (Gy)	37.15 (34.81-39.40)	37.54 (36.45-38.65)	37.60 (34.71-38.44)	1.000	1.000	1.000
Dmax (Gy)	43.15 (41.56-44.50)	42.43 (41.49-43.84)	42.02 (40.25-42.76)	1.000	.218	.166
Body						
Dmax (%)	106.29 (105.80-106.85)	106.50 (106.20-106.98)	106.19 (105.81-106.77)	<.001	1.000	<.001
V105 (cm^3^)	0.97 (0.41-2.40)	1.04 (0.67-2.71)	0.31 (0.22-0.90)	1.000	<.001	<.001
Delivery time (s)	124.00 (122.75-127.00)	157.00 (156.00-158.00)	151.00 (138.00-159.25)	.002	<.001	<.001
Total MU	588.98 (529.48-677.75)	521.15 (487.35-599.79)	773.50 (725.50-811.25)	.002	<.001	<.001

*Abbreviations:* CI = conformity index;C-VMAT = coplanar volumetric modulated arc therapy; Dmax = maximum dose; Dmean = mean dose; DSA = dynamic swing arc; DX = dose received by the X% of the volume; Gy = gray; HI = homogeneity index; IQR = interquartile range; MU = monitor unit NC-VMAT = noncoplanar volumetric modulated arc therapy; PTV = planning target volume; VX = the percentage of the organ volume that received by X Gy or more.

⁎ Wilcoxon signed-rank test was used with Bonferroni correction for multiple testing.

### Sparing of organs at risk

Table 4 summarizes the dose indices for the OARs in the 3 plans. The DSA plans significantly reduced the mean ipsilateral parotid gland dose by 5.78 Gy and 2.88 Gy compared to the C-VMAT and NC-VMAT plans (*P* < .001, *P* < .001, respectively). On the contralateral side, the DSA plans also significantly reduced the mean parotid gland dose by 6.93 Gy and 1.56 Gy (median) compared to the C-VMAT and NC-VMAT plans (*P* < .001, *P* < .001, respectively). For the ipsilateral and contralateral sides, NC-VMAT plans significantly reduced the mean parotid gland dose by 2.90 and 5.37 Gy (median) compared to C-VMAT plans (*P* < .001 and *P* < .001, respectively). For the oral cavity, the NC-VMAT and DSA plans significantly reduced the mean dose by 2.16 and 3.22 Gy (median) compared to the C-VMAT plans (*P* < .001, *P* < .001, respectively*)*. However, there was no significant difference in the mean oral cavity dose between the NC-VMAT and DSA plans (*P* = .181). For PCM, DSA plans significantly reduced mean dose compared to NC-VMAT plans (*P* < .001), and there were no significant differences between the C-VMAT and NC-VMAT plans and the C-VMAT and DSA plans. There were no significant differences in the maximum dose indices (Dmax and D1cc) of the brain, bilateral brachial plexuses, spinal cord, and mandible among the 3 plans. For the brain stem, the Dmax values in the NC-VMAT plans were higher than those in the C-VMAT and DSA plans *(P* = .001 and *P* = .005, respectively). However, there was no difference in the brainstem Dmax between the C-VMAT and DSA plans (*P* = .054). Although all the doses in the 3 plans were low for the eyes and lenses, the NC-VMAT plan had the highest Dmax, followed by the DSA and C-VMAT plans. For the right and left inner ears, there was a significant difference only between the C-VMAT and DSA plans (*P* = .034 and *P* < .001, respectively). For the cochlea, the 2 noncoplanar plans significantly reduced the median mean dose compared to the C-VMAT plans, except for the left cochlear dose in NC-VMAT. For the bilateral lung apexes, the DSA plan had the lowest median mean dose, followed by the NC-VMAT and C-VMAT plans. Regarding the overdose region, there were no significant differences between the DSA and C-VMAT plans in terms of the maximum dose to the body (*P* = 1.000). In contrast, NC-VMAT had worse Dmax values than C-VMAT and DSA plans (*P* < .001 and *P* < .001, respectively). DSA plans significantly reduced V105% compared with the C-VMAT and NC-VMAT plans (*P* < .001 and *P* < .001, respectively).

Furthermore, to assess the value of DSA plans for large tumors, a subgroup analysis of cases with a high-risk CTV > 150 cc (n = 16) was performed (Table E2). Even in cases with a larger high-risk CTV, the DSA plans significantly reduced the mean parotid gland dose by 6.92 and 4.02 Gy (median) on the ipsilateral side compared with the C-VMAT and NC-VMAT plans (*P* = .002 and *P* = .009, respectively). Also, the DSA plans significantly reduced the mean parotid gland dose by 6.69 and 2.41 Gy (median) on the contralateral side compared with C-VMAT and NC-VMAT plans (*P* = .001, *P* = .001, respectively). The proportion of patients who achieved the dose constraint with at least 1 parotid gland reduced to < 26 Gy was the highest in the DSA plans, followed by the NC-VMAT and C-VMAT plans (97% vs 91% vs 34%). The proportions of the above constraints achieved in both parotid glands were 69%, 41%, and 22% in DSA, NC-VMAT, and C-VMAT plans, respectively.

### NTCP for xerostomia and dysphagia

The DSA plans had the lowest median NTCP for xerostomia, followed by the NC-VMAT and C-VMAT plans (Table E3). In pairwise comparisons, 2 noncoplanar (NC-VMAT and DSA) plans significantly reduced NTCP for xerostomia compared with C-VMAT (*P* < .001 and *P* < .001, respectively). Furthermore, DSA plans significantly reduced NTCP for xerostomia compared with NC-VMAT plans (*P* < .001). For dysphagia, the NC-VMAT plan had the highest NTCP among the 3 plans. In pairwise comparisons, DSA plans significantly reduced NTCP for dysphagia compared with NC-VMAT (*P* < .001), but no significant difference was observed between C-VMAT and NC-VMAT plans, and C-VMAT and DSA plans (*P* = .168 and *P* = 1.000, respectively).

### Delivery time and MU values

Table 4 summarizes the MU values and delivery times in the 3 plans. In the C-VMAT plans, 30 of the 32 cases were created using 2 arcs and the remaining 2 cases were created using 3 arcs. In C-VMAT plans, the median delivery times of the 2 and 3 arcs were 124.50 and 182.50 seconds, respectively. The DSA plan increased the median delivery time by 16.6% (27.00 seconds) compared with the C-VMAT plan, with a significant difference (151.00 vs 124.00 seconds, *P* < .001). In contrast, the delivery times in the DSA plans were shorter than those in the NC-VMAT plans (*P* < .001). The total MU in the DSA plans was higher than that in the C-VMAT and NC-VMAT plans (*P* < .001 and *P* < .001, respectively).

## Discussion

To the best of our knowledge, this study is the first to evaluate the utility of DSA, a noncoplanar VMAT technique using the novel linear accelerator OXRAY, for HNSCC. To investigate the benefits of DSA, we performed a dosimetric comparison between DSA and conventional C-VMAT/NC-VMAT. In this study, the DSA plans significantly reduced doses to the parotid glands and oral cavity while maintaining target coverage compared to the conventional C-VMAT plans. Furthermore, compared with conventional NC-VMAT plans, DSA plans significantly reduced doses to the parotid gland and delivery time.

In RT for HNSCC, the radiation dose to the parotid glands has a direct impact on side effects such as xerostomia, requiring a strong dose reduction.^17^ Avraham et al^18^ reported that parotid glands receiving a mean dose > 26 Gy produced little saliva with no recovery over time. Therefore, dose reduction of the parotid glands is important to improve the quality of life of patients. However, when the high-risk CTV is extensive or close to the parotid glands or oral cavity, reducing the dose is often difficult with conventional coplanar VMAT. Magnuson et al^19^ reported an increase in a parotid dose of 2.0, 4.5, and 8.5 Gy, with increases in high-risk CTV of 4.1, 7.4, and 12.5 mm, respectively. In our study, in the group of patients with a high-risk CTV > 150 cm^3^, the mean doses for the ipsilateral and contralateral parotid were 29.57/24.42 Gy in C-VMAT plans, 26.67/20.14 Gy in NC-VMAT plans, and 22.65/17.73 Gy in DSA plans, respectively. As for those who achieved the clinical goal for the parotid glands (at least 1 side < 26 Gy), 11 of 32 cases (34%) failed to achieve it with C-VMAT plans, whereas almost all cases achieved it with NC-VMAT (29 of 32) and DSA (31 of 32) plans. Moreover, DSA reduced the bilateral parotid dose to < 26 Gy in 21 of 32 cases (66%) (Table E2). Additionally, there were significant differences in the NTCP for xerostomia among the 3 plans (Table E3). Our results imply that noncoplanar irradiation may reduce the probability of xerostomia compared to coplanar irradiation, especially in the DSA technique. In contrast, for PCM, the differences in the mean dose and NTCP value between the 3 plans were small. Therefore, the dose differences in PCM between these plans may not be clinically meaningful.

The mechanisms underlying our finding that the DSA plan reduced the doses to OARs while maintaining target coverage are as follows: First, DSA enables continuous noncoplanar irradiation using O-ring swing in synchronization with the gantry rotation. For head and neck cancers, previous studies have reported that noncoplanar irradiation improves target coverage and reduces the dose to OARs.^10^^,^^11^ Literature that verified the dosimetric benefits of the noncoplanar dynamic technique reported that the robustness and treatment of the noncoplanar dynamic technique are comparable to those of conventional coplanar VMAT.^11^ OXRAY is a newly developed linear accelerator whose DSA technique enables flexible noncoplanar irradiation because of the O-ring gantry swing. Furthermore, OXRAY has a 20 × 20-cm irradiation field, making it applicable to the head and neck region, which has not been addressed by noncoplanar VMAT using Vero4DRT. Second, the short-width high-speed drive MLC plays an important role in DSA plans using OXRAY. Several studies have reported that high-speed MLC drives dose reduction for the parotid glands and superior dose coverage for PTV compared to coplanar VMAT for bulky HNSCC.^20^^,^^21^ In DSA plans using OXRAY, the combination of O-ring swing and high-speed drive MLC may have improved dosimetric quality. The 2-arc DSA plans increased the median delivery time by 22 seconds (17%) compared to the 2-arc C-VMAT plans. However, the median delivery time of the 3-arc C-VMAT plans was longer than that of 2-arc DSA plans by 35 seconds (24%). In the DSA plans, the use of noncoplanar trajectories resulted in a longer delivery time than conventional coplanar VMAT for the same number of arcs. However, the difference is considered as small as possible because OXRAY is equipped with the O-ring gantry that can swing simultaneously with the gantry rotation. On the other hand, DSA plans significantly reduced delivery time compared to the 2-arc NC-VMAT plans.

This study has several limitations. First, C-VMAT plans were created by multiple planners in clinical practice, whereas NC-VMAT and DSA plans were created using a single planner. Therefore, the planners’ ability to plan the treatment may have affected the results of this study. Second, this study did not sufficiently consider the optimal settings for the DSA plans. In DSA plans, the planner manually confirmed the overlap between the OARs (particularly the parotid gland) and the target in the beam's eye view and selected the gantry trajectory specified by the O-ring angle and modulation point to minimize the overlap. Although the overlap between the target and OAR varied from case to case, the same gantry trajectory was adopted for all cases in the DSA plans. Therefore, the optimal gantry trajectory for each case was not investigated in this study. Moreover, we created DSA plans using an O-ring swing of up to 20°. However, the O-ring gantry of OXRAY allows a maximum swing of up to 60°. Further verification with various swing angles and modulation settings is required. Third, the field size of OXRAY (20 × 20 cm) may not be large enough to encompass all head and neck cancers. Although we could cover the targets of all patients using a 20 × 20-cm field in this study, some cases will be difficult to manage with this field size. Although not used in this study, OXRAY has a gimbal mechanism that allows the beam to move in the PAN and TILT directions, leading to a field expansion of 30 × 30 cm. Therefore, the gimbal mechanism allows irradiation of head and neck cancers requiring larger field sizes. Fourth, the dose to the hippocampus could not be evaluated in this study because we do not perform routine brain magnetic resonance imaging scans at our institute. Fifth, we created NC-VMAT and DSA plans as simulations, so the delivery times were estimated. In our next study, the delivery times will be measured.

## Conclusions

This study of 32 HNSCC patients revealed that DSA plans using OXRAY effectively lowered the mean radiation dose to the parotid glands and oral cavity. These plans successfully maintained PTV coverage while requiring longer delivery times compared to conventional coplanar VMAT approaches. When contrasted with conventional noncoplanar VMAT techniques, the DSA plans achieved both reduced parotid gland doses and shorter delivery times.

## Disclosures

Kento Tomizawa reports a relationship with Hitachi Ltd that includes: speaking and lecture fees. Shunta Jinno is an employee of Hitachi High-Tech Corporation, Tokyo, Japan. If there are other authors, they declare that they have no known competing financial interests or personal relationships that could have appeared to influence the work reported in this paper.

## Appendix Supplementary materials

Supplementary_Tables Image, application 1

## Footnotes

Supplementary material associated with this article can be found in the online version at doi:10.1016/j.adro.2024.101706.
