# Supplementary material for: Dosimetric Comparison of Noncoplanar VMAT Without Rotating the Patient Couch Versus Conventional Coplanar/Noncoplanar VMAT for Head and Neck Cancer: First Report of Dynamic Swing Arc
Source: Adv Radiat Oncol. 2024 Dec 30;10(3):101706. doi: 10.1016/j.adro.2024.101706 (PMC11794065; doi:10.1016/j.adro.2024.101706)
Supplement: Supplementary_Tables [file mmc1.docx]

Supplementary Table E1 Parameters used for normal tissue complication evaluation.

| Critical structure |  | Endpoint | m | n | TD_50_ (Gy) |
| --- | --- | --- | --- | --- | --- |
| Xerostomia |  | ≥ 2 levels of increase from baseline in the patient reported toxicity scale at 6 months | 0.53 | 1 | 31.4 |
| Dysphasia |  | Acute dysphagia ≥ Grade 2 | 1.129 | 1 | 30.5 |

Abbreviations: NTCP, normal tissue complication; TD_50_, 50% toxic dose.

Supplementary Table E2 Dose index for PTVs and parotid glands in cases with high-risk CTV volumes larger than 150 cc between coplanar/non-coplanar VMAT and DSA plans

|  |  |  | C-VMAT | NC-VMAT | DSA |  | *P* values* | | |
| --- | --- | --- | --- | --- | --- | --- | --- | --- | --- |
|  |  |  | Median (IQR) | Median (IQR) | Median (IQR) |  | C-VMAT vs. NC-VMAT | C-VMAT vs. DSA | NC-VMAT vs. DSA |
| High-risk PTV | |  |  |  |  |  |  |  |  |
|  | D98 (%) |  | 95.12 (93.94–96.04) | 96.16 (94.68–97.00) | 96.04 (94.81–96.78) |  | 0.001 | 0.084 | 0.001 |
|  | CI |  | 0.96 (0.95–0.97) | 0.97 (0.95–0.98) | 0.98 (0.97– 0.98) |  | 1.000 | 0.010 | 0.011 |
|  | HI |  | 9.46 (8.63–10.36) | 8.04 (7.26–9.94) | 8.11 (7.26–9.41) |  | 0.140 | 0.031 | 1.000 |
| Low-risk PTV | |  |  |  |  |  |  |  |  |
|  | D98 (%) |  | 95.04 (94.10–96.44) | 95.30 (93.07–96.22) | 96.63 (94.63–97.49) |  | 0.001 | 0.056 | 0.001 |
| Parotid ipsilateral | |  |  |  |  |  |  |  |  |
|  | Dmean (Gy) |  | 29.57 (27.52–40.31) | 26.67 (23.33–35.91) | 22.65 (17.71–33.29) |  | 0.005 | 0.002 | 0.009 |
| Parotid contralateral | |  |  |  |  |  |  |  |  |
|  | Dmean (Gy) |  | 24.42 (22.89–27.74) | 20.14 (14.16–23.90) | 17.73 (12.39–20.24) |  | 0.003 | 0.001 | 0.001 |

Abbreviations: C-VMAT, coplanar volumetric modulated arc therapy; NC-VMAT, non-coplanar volumetric modulated arc therapy; DSA, dynamic swing arc; IQR, interquartile range; Gy, gray; PTV, planning target volume; DX, dose received by the X% of the volume; CI, Conformity Index; HI, Homogeneity Index.

*Wilcoxon signed rank test was used by the Bonferroni correction for multiple testing

Supplementary Table E3 Comparison of normal tissue complication between coplanar/non-coplanar VMAT and DSA plans

|  |  | C-VMAT | NC-VMAT | DSA |  | *P* values* | | |
| --- | --- | --- | --- | --- | --- | --- | --- | --- |
|  |  | Median (IQR) | Median (IQR) | Median (IQR) |  | C-VMAT vs. NC-VMAT | C-VMAT vs. DSA | NC-VMAT vs. DSA |
| Xerostomia |  | 0.44 (0.39–0.51) | 0.36 (0.26–0.43) | 0.31 (0.23–0.37) |  | < 0.001 | < 0.001 | < 0.001 |
| Dysphasia |  | 0.76 (0.73–0.79) | 0.77 (0.73–0.79) | 0.76 (0.72–0.77) |  | 0.168 | 1.000 | 0.001 |

Abbreviations: C-VMAT, coplanar volumetric modulated arc therapy; NC-VMAT, non-coplanar volumetric modulated arc therapy; DSA, dynamic swing arc; IQR, interquartile range.

*Wilcoxon signed rank test was used by the Bonferroni correction for multiple testing
